# Phosphorylation of TFIIB Links Transcription Initiation and Termination

**DOI:** 10.1016/j.cub.2010.01.052

**Published:** 2010-03-23

**Authors:** Yuming Wang, Jennifer A. Fairley, Stefan G.E. Roberts

**Affiliations:** 1 Faculty of Life Sciences, University of Manchester, Oxford Road, Manchester M13 9PT, UK; 2 Department of Biological Sciences, University at Buffalo (The State University of New York), Cooke Hall, Buffalo, NY 14260, USA

**Keywords:** DNA

## Abstract

The general transcription factor TFIIB plays a central role in preinitiation complex (PIC) assembly and the recruitment of RNA polymerase II (RNA pol II) to the promoter [1]. Recent studies have revealed that TFIIB engages in contact with the transcription termination region and also with complexes that are involved in 3′ end processing and/or termination [2–9]. Here we report that TFIIB can be phosphorylated within the N terminus at serine 65 in vivo and that the phosphorylated form of TFIIB is present within (PICs). Surprisingly, TFIIB serine 65 phosphorylation is required after the phosphorylation of serine 5 of RNA pol II C-terminal domain (CTD) has occurred, but before productive transcription initiation begins. We show that phosphorylation of TFIIB at serine 65 regulates the interaction between TFIIB and the CstF-64 component of the CstF 3′ cleavage and polyadenylation complex. This directs the recruitment of CstF (cleavage stimulatory factor) to the terminator and also the recruitment of the CstF and CPSF (cleavage and polyadenylation specific factor) complexes to the promoter. Our results reveal that phosphorylation of TFIIB is a critical event in transcription that links the gene promoter and terminator and triggers initiation by RNA pol II.

## Highlights

► TFIIB is phosphorylated at serine 65 in vivo ► Phosphorylation of TFIIB serine 65 is important for transcription in vivo ► TFIIB phosphorylation is required after RNA pol II CTD serine 5 phosphorylation ► Phosphorylation of TFIIB serine 65 links the promoter and terminator

## Results and Discussion

### Phosphorylation of TFIIB In Vitro

**Figure 1:**
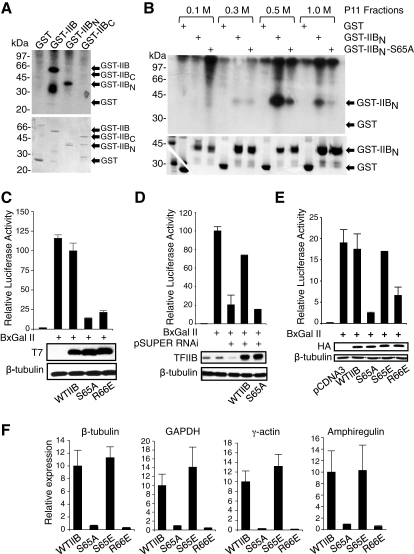
Phosphorylation of TFIIB Serine 65 In Vitro and the Effects of Serine 65 Substitutions on Transcription In Vivo (A) One microgram each of recombinant GST, GST-IIB, or derivatives GST-IIB_N_ (residues 1–124) or GST-IIB_C_ (residues 124–316) linked to glutathione agarose beads prepared as described previously [26] were incubated with [γ-^32^P]ATP and crude HeLa cell nuclear extract. Following the kinase reaction, the beads were washed and separated by SDS-PAGE, the gel was stained with Coomassie blue and dried, and ^32^P incorporation was then visualized by autoradiography. GST-IIB, GST-IIB_C_, GST-IIB_N_, and GST are indicated at right. The truncation product of the GST-IIB fusion protein, which likely contains the N terminus of TFIIB, also incorporated the radiolabel. (B) HeLa cell nuclear extract was subject to P11 chromatography, with step elution at 0.1, 0.3, 0.5, and 1.0 M KCl. The fractions (labeled above the lanes) were incubated with recombinant GST, GST-IIB_N_, or GST-IIB_N_ S65A in the presence of [γ^32^P]ATP and analyzed as in (A). (C) HEK293T cells were transfected with 1 μg of AdML luciferase reporter (which contains five GAL4 DNA-binding sites), 1 μg of plasmid driving expression of BxGalII (GAL4 residues 1–147 linked to the region II activation domain of GAL4), and 500 ng of vector driving expression of T7-tagged wild-type TFIIB or the mutant derivatives R66E and S65A. Forty-eight hours later, cell lysates were prepared and luciferase activity was measured, or western blotting with anti-T7 and β-tubulin antibodies was performed (below). Data are representative of at least three independent experiments performed in triplicate. (D) Cells were transfected with 1 μg pSUPER RNAi and 500 ng of vector driving expression of the indicated TFIIB derivatives and analyzed as in (C), except that anti-TFIIB antibodies were used in the immunoblot. (E) As in (C), except that TFIIB mutant derivative S65E was also included in the assay, HA-tagged TFIIB derivatives were employed, and anti-HA antibodies were used in the immunoblot. (F) HEK293T cells were transfected with vector driving expression of wild-type TFIIB or the indicated TFIIB mutant derivatives along with a vector driving expression of GFP. The transfected cells were selected by fluorescence-activated cell sorting (FACS) after 48 hr, and total RNA was extracted. Quantitative reverse transcriptase-polymerase chain reaction (qRT-PCR) was performed to detect β*-tubulin*, *GAPDH*, γ*-actin*, and *amphiregulin* mRNA levels relative to the polymerase I (pol I) *18S* transcript. Error bars denote standard deviation (SD).

We tested whether human TFIIB could be phosphorylated when incubated with a crude HeLa cell nuclear extract. GST, GST-TFIIB (GST-IIB), GST-IIB_N_ (residues 1–124), and GST-IIB_C_ (residues 124–316) were expressed in *E. coli* and purified by binding to glutathione agarose beads. The GST fusion proteins, linked to beads, were incubated with HeLa nuclear extract in the presence of [γ-^32^P]ATP. The beads were washed extensively, and proteins were released by heating to 95°C in SDS-PAGE loading dye, resolved by SDS-PAGE followed by staining with Coomassie blue, and then subjected to autoradiography. Figure 1A shows the autoradiogram (above) and the same Coomassie-stained gel (below). The GST-IIB fusion protein, but not GST, incorporated the radiolabeled phosphate. Analysis of the GST-IIB_N_ and GST-IIB_C_ derivatives demonstrated that the radiolabel was exclusively incorporated into the N-terminal 124 residues of TFIIB, and not into the C-terminal core domain.

DNA-PK has previously been reported to phosphorylate TFIIB residue serine 65 in vitro [10]. We therefore produced a GST-TFIIB (1–124) derivative in which serine 65 had been substituted with alanine (S65A). GST, GST-IIB_N_, and GST-IIB_N_ S65A were incubated with the 0.1, 0.3, 0.5, and 1.0 M salt fractions derived from phosphocellulose chromatography of HeLa nuclear extract (Figure 1B). The TFIIB kinase activity present in the 0.5 M P11 fraction (which contains the major TFIIB kinase activity) and the 1.0 M P11 fraction showed a significant reduction in kinase activity toward the TFIIB S65A derivative when compared with wild-type TFIIB, suggesting that serine 65 might be a major site of the phosphorylation.

### TFIIB Serine 65 Is Required for Transcription In Vivo

We used transient transfection of human embryonic kidney 293T (HEK293T) cells to analyze the effect of ectopic expression of a TFIIB S65A mutant derivative on the activity of a luciferase reporter plasmid linked to the adenovirus major late (AdML) core promoter under the control of the activator BxGalII. Expression of wild-type TFIIB did not significantly affect transcription of the reporter. As we have reported previously, the mutant TFIIB derivative R66E inhibited transcription ([11, 12]; Figure 1C). Similarly, the TFIIB S65A mutant derivative inhibited transcription. Immunoblotting with anti-T7 antibodies (to detect an epitope tag at the C terminus of the ectopically expressed TFIIB) confirmed the equivalent expression of the TFIIB derivatives.

We next employed an RNA interference (RNAi)-based approach described by us previously to analyze the function (or functions) of the TFIIB mutant derivatives in living cells in the absence of endogenous TFIIB [12]. Vector-derived RNAi was used to ablate the expression of endogenous TFIIB while, simultaneously, wild-type TFIIB or TFIIB S65A that contains a silent mutation within the RNAi target sequence was introduced. Immunoblotting with anti-TFIIB antibodies confirmed the RNAi-mediated knockdown of TFIIB and equivalent expression of wild-type TFIIB and TFIIB S65A (Figure 1D). The expression of wild-type TFIIB restored reporter activity but, consistent with its inhibitory activity, expression of TFIIB S65A did not.

Next, we tested the effect of ectopic expression of the potential phosphomimetic TFIIB mutant derivative S65E on transcription of the AdML reporter. As before, expression of TFIIB S65A was a potent inhibitor of AdML reporter activity (Figure 1E). However, expression of the TFIIB S65E mutant derivative elicited an effect similar to that observed with wild-type TFIIB, suggesting that TFIIB S65E is a phosphomimetic protein that can support transcription.

We next analyzed a selection of endogenous genes to determine whether their activity was affected by the expression of the TFIIB derivatives. Plasmids driving expression of wild-type TFIIB or the mutant TFIIB derivatives were transfected into 293T cells along with a plasmid driving expression of green fluorescent protein (GFP). Forty-eight hours later, the transfected cells were separated and collected by fluorescence-activated cell sorting (FACS), and the expression levels of β*-tubulin*, *GAPDH*, γ*-actin*, and *amphiregulin* were analyzed by quantitative polymerase chain reaction, normalized against the RNA polymerase I (pol I) *18S* transcript (Figure 1F). Expression of TFIIB S65A or TFIIB R66E resulted in the inhibition of all of the pol II transcripts that we tested, but the phosphomimetic TFIIB S65E did not.

### The TFIIB Mutant Derivatives Are Defective at One or More Events following Preinitiation Complex Assembly

**Figure 2:**
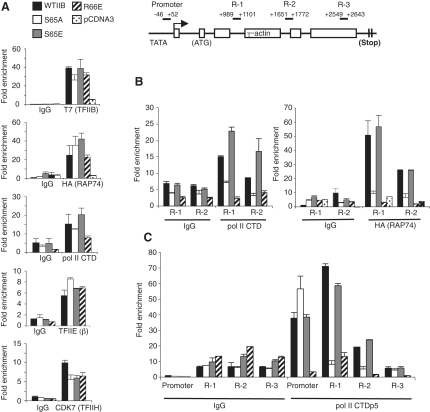
TFIIB B Finger/B Reader Mutant Derivatives Are Able to Support Preinitiation Complex Assembly but Are Defective in Supporting the Transition of Pol II to a Productive Transcription Complex (A) Left: HEK293T cells were transfected with pcDNA3 vector, pcDNA3 driving expression of T7-tagged wild-type TFIIB, or the mutant derivatives indicated. Vector driving expression of HA-tagged RAP74 was cotransfected into the cells where indicated. For endogenous pol II, TFIIE, and TFIIH analysis, cells were cotransfected with a plasmid driving GFP and processed as described in the text. Chromatin immunoprecipitation (ChIP) was performed as described previously [27] with modifications (see Supplemental Experimental Procedures) with the antibodies indicated, and data are presented as fold enrichment of γ*-actin* promoter DNA over nonpromoter DNA of the same gene or the pol I-transcribed *18S* gene in the immunoprecipitation. Right: schematic diagram of the γ*-actin* gene showing the TATA box/promoter region, open reading frames (boxes), and terminator (vertical lines). The positions of real-time PCR products used in the ChIP analysis are marked relative to the transcription start site. (B) ChIP analysis was performed with anti-pol II C-terminal domain (CTD) (left) or anti-HA (TFIIF, RAP74; right) antibody, and PCR analysis of immunoprecipitated chromatin was performed on coding regions 1 and 2 of the γ*-actin* gene. (C) ChIP of phosphorylated CTD at serine 5 (CTDp5) was performed as in (B), except that S5-P-CTD antibody was used in the immunoprecipitation. Error bars denote SD. See also Figures S1 and S2.

Chromatin immunoprecipitation (ChIP) was employed to analyze the effect of TFIIB serine 65 substitution on the assembly of TFIIB at the γ*-actin* promoter in vivo. Empty expression vector or vector driving expression of T7 epitope-tagged wild-type TFIIB or the derivatives was transfected into 293T cells, and 48 hr later, ChIP was performed with either anti-T7 tag antibodies or control mouse IgG antibodies. The promoter region of the γ*-actin* gene and also a control nonpromoter region were amplified, and the data were expressed as fold enrichment of the γ*-actin* promoter relative to the nonpromoter region (Figure 2A). The results show that TFIIB S65A, S65E, and R66E were recruited to the γ*-actin* promoter to a level similar to that observed with wild-type TFIIB.

We next analyzed the effect of the TFIIB mutant derivatives on the recruitment of TFIIF to the γ*-actin* promoter. To ensure that TFIIF recruitment was only analyzed in transfected cells, along with the TFIIB expression plasmids we cotransfected a plasmid driving expression of a HA-tagged RAP74 subunit of the TFIIF complex. Expression of the HA-tagged RAP74 was confirmed by immunoblotting whole-cell extracts with anti-HA antibodies (see Figure S1A available online). ChIP was performed with either anti-HA antibodies or a control antibody, and the data are presented as fold enrichment of the γ*-actin* promoter relative to the pol I-transcribed *18S* gene (Figure 2A, second panel from top). HA-tagged RAP74 was recruited to the γ*-actin* promoter in the presence of all of the transfected TFIIB derivatives.

We next tested whether TFIIB serine 65 is important for pol II recruitment to promoters, using the GFP/FACS protocol described above to harvest only the transfected cells. ChIP was performed with anti-pol II C-terminal domain (CTD) antibody or a control antibody. As observed with TFIIF, pol II was also recruited to the γ*-actin* promoter when the cells were transfected with wild-type TFIIB or the TFIIB mutant derivatives (Figure 2A). Using the same approach, we determined that both TFIIE (β subunit) and TFIIH (CDK7) were also recruited to the γ*-actin* promoter in the presence of all of the TFIIB mutant derivatives (Figure 2A). Comparable data were obtained when we analyzed the β*-tubulin* and *GAPDH* promoters (Figures S1A and S2A). Thus, TFIIB S65A and also TFIIB R66E manifest their transcriptional defects at an event following preinitiation complex (PIC) assembly.

We next performed ChIP analysis to examine the presence of pol II within the coding region of the γ*-actin* gene. Cells were transfected as above, and ChIP was performed with anti-pol II CTD antibodies. Primers were used to amplify two internal regions of the γ*-actin* gene (Figure 2B, left; primer locations are shown in schematic above). Pol II was readily detected within the coding region of the γ*-actin* gene when either wild-type TFIIB or TFIIB S65E was expressed. In contrast, and consistent with the transcription data, we were unable to detect pol II within the coding region when TFIIB S65A or TFIIB R66E was expressed. Translocation of TFIIF (as HA-tagged RAP74) into the γ*-actin* gene internal region was also blocked by expression of either TFIIB S65A or R66E (Figure 2B, right). Thus, TFIIB S65A and R66E can form PICs that contain pol II, TFIIF, TFIIE, and TFIIH but fail to form a productive transcription complex. Comparable effects were observed when we analyzed the β*-tubulin* and *GAPDH* genes (Figures S1B and S2B).

### The Integrity of TFIIB Serine 65 Is Required after Pol II CTD Serine 5 Phosphorylation

At transcription initiation, the pol II CTD is phosphorylated at serine 5 [13]. We therefore determined whether the TFIIB mutant derivatives support phosphorylation of serine 5 of the pol II CTD. Cells were transfected as above, and ChIP was performed with anti-phosphoserine 5 CTD antibodies (CTDp5). Primers were used to amplify four distinct regions across the γ*-actin* gene from the promoter to the termination region (Figure 2C). Expression of either wild-type TFIIB or TFIIB S65E supported phosphorylation of pol II CTD serine 5 at the promoter and within the early coding region of the γ*-actin* gene but then declined toward the 3′ region of the gene. These results are consistent with the known function of pol II CTD serine 5 phosphorylation mainly at, or proximal to, the promoter [13]. However, when we analyzed the TFIIB S65A and R66E mutant derivatives, we surprisingly found that they elicited distinct effects on pol II CTD serine 5 phosphorylation. The expression of TFIIB R66E caused a significant reduction in the detection of pol II serine 5 phosphorylation at the promoter and also the early coding region of the γ*-actin* gene, suggesting that the assembly of PICs containing TFIIB R66E prevents the phosphorylation of pol II CTD serine 5 even though TFIIH is present within the complex (see bottom panel of Figure 2A). The mechanistic basis for this effect is not clear, but it is possible that either TFIIH or pol II are incorrectly aligned within the PIC or that TFIIB can directly modulate the activity of TFIIH or perhaps other components of the PIC that augment CDK7 activity [14].

In contrast to the effect elicited by TFIIB R66E, expression of TFIIB S65A did not prevent the phosphorylation of pol II serine 5 at the promoter but significantly reduced its occupancy at the early coding region of the γ*-actin* gene. Comparable results were obtained when we examined the β*-tubulin* and *GAPDH* genes (Figures S1C and S2C). Thus, the TFIIB mutant derivatives R66E and S65A manifest their defects in supporting transcription at distinct events in the transcription sequence. PICs that form with TFIIB R66E are rendered defective prior to pol II CTD serine 5 phosphorylation. In contrast, complexes that contain TFIIB S65A undergo phosphorylation of pol II CTD serine 5, but the complexes are not transcriptionally productive.

### TFIIB Is Phosphorylated at Serine 65 In Vivo

**Figure 3:**
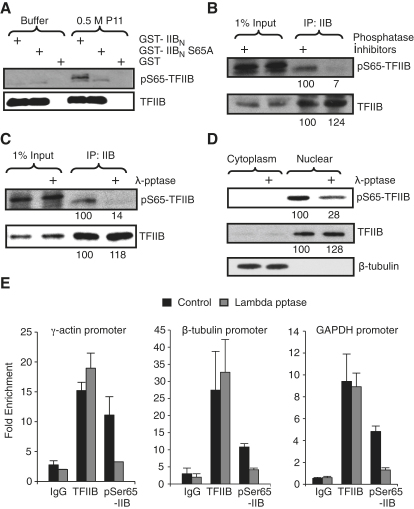
Phosphorylation of TFIIB at Serine 65 In Vivo and Localization at the Promoter (A) GST or the indicated GST-TFIIB derivatives were incubated with either 150 mM KCl-buffer D or the same buffer containing 0.5 M P11 fraction (as described in Figure 1). The kinase reaction was terminated by the addition of SDS buffer, and the samples were subjected to SDS-PAGE. Western blots were performed with either anti-pS65-TFIIB antibody or anti-TFIIB antibody. (B) Endogenous TFIIB was immunoprecipitated from HEK293T whole-cell extracts with anti-TFIIB antibodies. Phosphatase inhibitors were used where indicated (+) in the lysis and wash buffers. The immunoprecipitates were analyzed as in (A). Quantitation of select bands is shown below the autoradiogram. (C) Immunoprecipitation of endogenous TFIIB was performed in lysis buffer containing phosphatase inhibitors. After the final wash, the antibody-antigen complex was treated with lambda phosphatase (λ-pptase) where indicated (+) and then subjected to SDS-PAGE and blotting as in (A). (D) U2OS cells were separated into cytoplasmic and nuclear fractions. The fractions were then incubated with or without lambda phosphatase as indicated and subjected to SDS-PAGE. Western blots were performed with anti-TFIIB, anti-β-tubulin, or anti-pS65-TFIIB antibodies. (E) Sonicated chromatin was prepared from 293T cells and incubated with lambda phosphatase where indicated. ChIP was performed with general anti-TFIIB antisera, anti-pS65-TFIIB antibodies, or control antibodies. The data are presented as fold enrichment of γ*-actin*, β*-tubulin*, and *GAPDH* promoter DNA over nonpromoter DNA in the immunoprecipitation. Error bars denote SD.

We generated and purified antibodies against a TFIIB peptide containing phosphorylated serine 65 and then used immunoblotting to test their specificity. GST, GST-IIB_N_, and GST-IIB_N_ S65A linked to glutathione agarose beads were incubated with the 0.5 M fraction derived from fractionation of HeLa cell nuclear extract over a P11 column (as in Figure 1), or with buffer alone, in an in vitro kinase assay. Immunoblotting was then performed with either anti-TFIIB antibodies or with the purified antibodies raised against phosphoserine 65 (pS65-TFIIB; Figure 3A). The pS65-TFIIB antibody produced a robust signal against GST-IIB_N_, but not with the S65A mutant derivative, which only occurred when the fusion proteins had been incubated with the 0.5 M P11 fraction and not with buffer alone. Blotting the same samples with general TFIIB antisera produced a signal with both wild-type TFIIB and the S65A derivative that was not dependent on prior incubation of the fusion protein with the 0.5 M P11 fraction. Thus, the pS65-TFIIB antibodies react specifically with TFIIB only when phosphorylated at serine 65.

We next prepared cell lysates from 293T cells in the presence or absence of phosphatase inhibitors, immunoprecipitated the endogenous TFIIB, and then immunoblotted with either the pS65-TFIIB antibody or general anti-TFIIB antibody (Figure 3B). Immunoblotting with the general TFIIB antibody demonstrated equivalent immunoprecipitation of TFIIB regardless of the presence or absence of phosphatase inhibitors during the lysate preparation (quantitation is shown below the autoradiogram). In contrast, immunoblotting the same samples with the pS65-TFIIB antibody showed a significantly lower signal in the TFIIB immunoprecipitate derived from the preparation that lacked phosphatase inhibitors. Next, we prepared TFIIB immunoprecipitates as above and incubated the samples with lambda phosphatase (λ-pptase). This treatment did not affect the total TFIIB protein level but significantly reduced the signal with the anti-pS65-TFIIB antibodies (Figure 3C). Thus, the recognition of TFIIB by anti-pS65-TFIIB antibody is dependent on TFIIB phosphorylation in vivo.

We also used the anti-pS65-TFIIB antibodies to immunoblot nuclear and cytoplasmic extracts prepared in the presence of phosphatase inhibitors from U2OS cells. The extracts were incubated in the absence or presence of lambda phosphatase and then immunoblotted as before (Figure 3D). As expected, endogenous TFIIB was present exclusively in the nuclear fraction and β-tubulin in the cytoplasmic fraction. Immunoblotting with pS65-TFIIB antibody revealed a significant reduction in signal when the samples had been incubated with lambda phosphatase. Collectively, the data in Figures 3A–3D suggest that a portion of endogenous TFIIB in 293T cells and U2OS cells is phosphorylated at serine 65.

Next, we used ChIP to determine whether TFIIB phosphorylated at serine 65 is present within PICs. Following the fragmentation of chromatin derived from nontransfected 293T cells and prior to the addition of antibodies, the samples were incubated with buffer alone or with buffer containing lambda phosphatase. ChIP was then performed with control antibodies, general anti-TFIIB antibodies, or anti-pS65-TFIIB antibodies. The γ*-actin*, β*-tubulin*, and *GAPDH* promoters were then amplified, and the data are presented graphically as fold enrichment over the signal generated by amplification of a control internal region of each gene (Figure 3E). The ChIP signal generated by the general TFIIB antibodies was not affected by the treatment of the chromatin with lambda phosphatase. In contrast, ChIP of the three promoters by the anti-pS65 IIB antibodies was significantly reduced when the chromatin samples had been incubated with lambda phosphatase. Taken together, the data in Figure 3 suggest that a portion of TFIIB is phosphorylated at serine 65 in vivo and that this includes promoter-bound TFIIB.

### Phosphorylation of TFIIB Serine 65 Is Required for the Recruitment of 3′ Termination Complexes to the Promoter and Terminator

**Figure 4:**
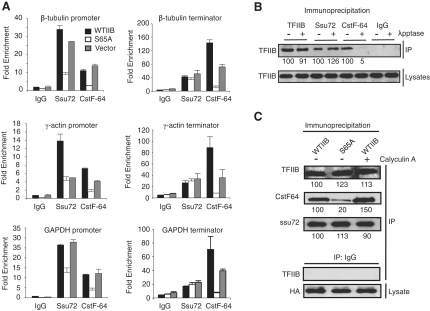
Recruitment of mRNA Processing Factors to the Promoter and Terminator Is Regulated by the Phosphorylation of TFIIB Residue Serine 65 (A) HEK293T cells were transfected with pcDNA3 vector, the same vector driving expression of wild-type TFIIB or TFIIB S65A, and vector driving expression of GFP. FACS sorting was used to select the transfected cells. ChIP analysis was performed with anti-Ssu72 antibody, anti-CstF-64 antibody, or control rabbit IgG. qPCR was performed, and data are presented as fold enrichment of specific β*-tubulin*, γ*-actin*, or *GAPDH* promoter DNA (left) or terminator (primer R-3, right) over the pol I-transcribed gene *18S* DNA. Error bars denote SD. (B) HEK293T whole-cell extract was prepared in the presence of phosphatase inhibitors and then treated with lambda phosphatase where indicated. The cell lysates were then subjected to immunoprecipitation with control antibodies or anti-TFIIB, anti-Ssu72, or anti-CstF-64 antibodies. Immunoprecipitates (top) and samples of the input lysates (bottom) were immunoblotted with anti-TFIIB antibody. (C) Top: HEK293T cells were transfected with vector driving expression of HA-tagged wild-type TFIIB or the TFIIB S65A mutant derivative. Forty-eight hours later, the cells were treated with 100 nM calyculin A where indicated for 30 min prior to lysis. Immunoprecipitation of the overexpressed TFIIB was performed with anti-HA antibody in buffer containing phosphatase inhibitors. Immunoblots with anti-TFIIB, anti-Ssu72, or anti-CstF-64 antibodies are shown. Below: the input lysates were immunoblotted with anti-HA antibody.

Our data so far suggest that phosphorylation of TFIIB serine 65 is important for one or more steps that occur after the phosphorylation of serine 5 of the pol II CTD. TFIIB has been linked with the CPSF (cleavage and polyadenylation specific factor) and CstF (cleavage stimulatory factor) complexes, which are recruited to the PIC and are required for cleavage and polyadenylation of mRNA [3, 4]. We therefore used ChIP to determine whether the recruitment of Ssu72 (a component of the CPSF complex) and CstF-64 (a component of the CstF complex) to the promoter or terminator was dependent on the integrity of TFIIB residue serine 65. 293T cells were transfected as above and then subjected to FACS, and ChIP was performed with anti-Ssu72 antibodies, anti-CstF-64 antibodies, or control antibodies. The promoter and terminator regions of the β*-tubulin*, γ*-actin*, and *GAPDH* genes were amplified, and the data were quantitated and expressed as fold enrichment over the signal generated by the amplification of a fragment of the *18S* gene (Figure 4A). Transfection of wild-type TFIIB resulted in robust recruitment of both Ssu72 and CstF-64 to all three promoter and terminator regions. When TFIIB S65A was overexpressed, the recruitment of both Ssu72 and CstF-64 to the promoter was considerably impaired. At the terminator, however, expression of TFIIB S65A significantly reduced occupancy by CstF-64, but not Ssu72.

Both Ssu72 and CstF-64 can interact directly with TFIIB [7, 8, 15, 16]. We therefore prepared whole-cell lysates from 293T cells and either mock incubated or incubated the extracts with lambda phosphatase. Immunoprecipitation was performed with anti-TFIIB, anti-Ssu72, anti-CstF-64, or control IgG antibodies and immunoblotted with general anti-TFIIB antibodies (Figure 4B, top). The anti-Ssu72 and anti-CstF-64 antibodies both coprecipitated TFIIB in the extracts that were mock treated. However, in the extracts that had been treated with lambda phosphatase, anti-Ssu72 antibodies coprecipitated TFIIB but the anti-CstF-64 antibodies did not. Immunoblotting with anti-TFIIB antibodies confirmed that similar levels of TFIIB were present in the extracts (Figure 4B, bottom). Thus, the interaction between Ssu72 and TFIIB is phosphorylation independent, but the interaction between TFIIB and CstF-64 is dependent on phosphorylation. Next, we examined the effect of TFIIB serine 65 substitution with alanine on the association of TFIIB with Ssu72 and CstF-64 by coimmunoprecipitation. 293T cells were transfected with plasmids driving expression of either HA-tagged wild-type TFIIB or HA-tagged TFIIB S65A. Whole-cell extracts were prepared in the presence of phosphatase inhibitors from untreated cells or cells that had been treated for 30 min with the general serine/threonine phosphatase inhibitor calyculin A. The ectopically expressed TFIIB was immunoprecipitated with anti-HA antibodies, and the samples were immunoblotted with anti-Ssu72, anti-CstF-64, or general anti-TFIIB antibodies (Figure 4C). Although both wild-type TFIIB and the S65A derivative showed equivalent immunoprecipitation with general anti-TFIIB antibodies, there was a significant reduction of CstF-64 coimmunoprecipitation in the lysate containing TFIIB S65A. Furthermore, the coimmunoprecipitation of CstF-64 with wild-type TFIIB was enhanced by calyculin A. In contrast, the coimmunoprecipitation of Ssu72 with TFIIB was not affected by S65A substitution or calyculin A. Taken together, the data in Figure 4 show that recruitment of CstF-64 and Ssu72 to the promoter is dependent upon TFIIB phosphorylation at residue serine 65, while the recruitment of CstF-64, but not Ssu72, is specifically affected at the terminator. This is due at least in part to the modulation of the TFIIB-CstF-64 interaction by phosphorylation of TFIIB serine 65. The data also suggest that TFIIB-mediated recruitment of CstF to the promoter and/or terminator is a prerequisite for the recruitment of CPSF to the promoter, but not the terminator.

Purified DNA-PK can phosphorylate TFIIB serine 65 in vitro, but there is no evidence as yet that DNA-PK is a physiologically relevant TFIIB kinase [10]. Our attempts to purify the kinase from the 0.5 M P11 fraction (see Figure 1) resulted in loss of activity. However, the CDK inhibitor olomoucine was able to inhibit the in vitro kinase reaction (Figure S3A). In addition, the CDK inhibitor DRB at a 200 μM concentration (to which the CDK7 activity of TFIIH is sensitive) inhibited phosphorylation of TFIIB serine 65 in vitro and in vivo (Figures S3B–S3E). These data suggest that TFIIH might be the TFIIB serine 65 kinase. However, purified TFIIH failed to phosphorylate TFIIB in vitro (data not shown), suggesting that other factors are required.

Previous studies suggested that gene looping, which juxtaposes promoters and terminators, is dependent on both TFIIB and components of the CPSF and CstF complexes [8, 17–19]. Serine 65 is within a region of TFIIB termed the B finger or B reader that is essential for these activities and also plays a role in transcription start site selection, bubble formation, and promoter clearance [1, 20–23]. Our current data show that the phosphorylation of TFIIB serine 65 is required for the efficient recruitment of CstF to the terminator and the recruitment of both CPSF and CstF to the promoter at a stage following phosphorylation of serine 5 of the pol II CTD, but before the transition of pol II into a productive transcribing enzyme. These observations suggest that phosphorylation of TFIIB serine 65 is important for the formation of gene loops and potentially forms a critical trigger for the initiation of transcription. We also note that the TFIIB serine 65 derivatives do not alter transcription start site selection (data not shown).

The TFIIB B finger/B reader plays a role in modulating the conformation of TFIIB [1, 11, 24]. It is therefore possible that phosphorylation of TFIIB serine 65 can alter the conformation of TFIIB and its capacity to engage in contacts with other components that are recruited to the PIC, for example CstF-64. In this regard, it is noteworthy that transcriptional activators can induce a conformational change in TFIIB, raising the possibility that phosphorylation of TFIIB serine 65 also forms part of the transcriptional activation process [1, 11, 24, 25].

## Supplemental Information

Document S1. Three Figures, Supplemental Experimental Procedures, and One Table
