# Supplementary material for: Phosphorylation of TFIIB Links Transcription Initiation and Termination
Source: Curr Biol. 2010 Mar 23;20(6):548–53. doi: 10.1016/j.cub.2010.01.052 (PMC2849011; doi:10.1016/j.cub.2010.01.052)
Supplement: Document S1. Three Figures, Supplemental Experimental Procedures, and One Table [file mmc1.pdf]

Current Biology, Volume *20*

Supplemental Information

Phosphorylation of TFIIB Links Transcription Initiation and Termination

Yuming Wang, Jennifer A. Fairley, and Stefan G. E. Roberts

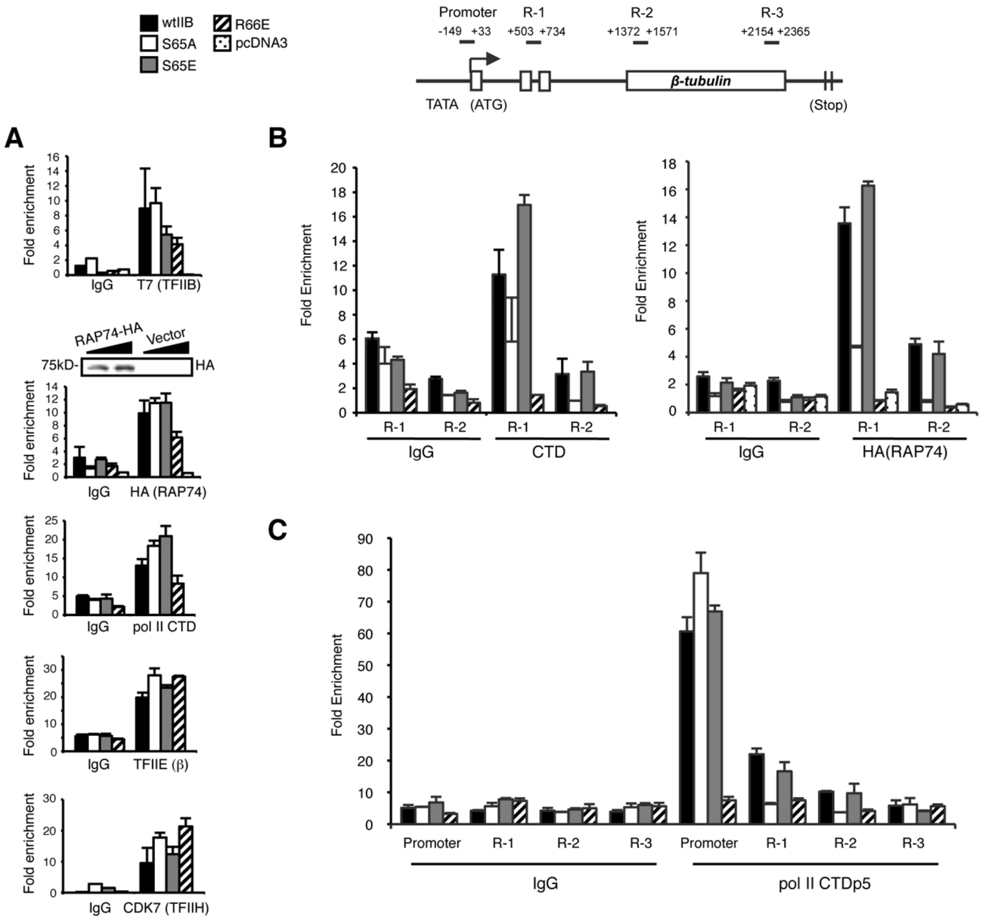

Figure S1. Analysis of PIC Assembly, Pol II localization, and Pol II CTD Phosphorylation within the β-*tubulin* Gene, Related to Figure 2

Data were generated and analyzed as in Figure 2, except that the subject was the β-*tubulin* gene.

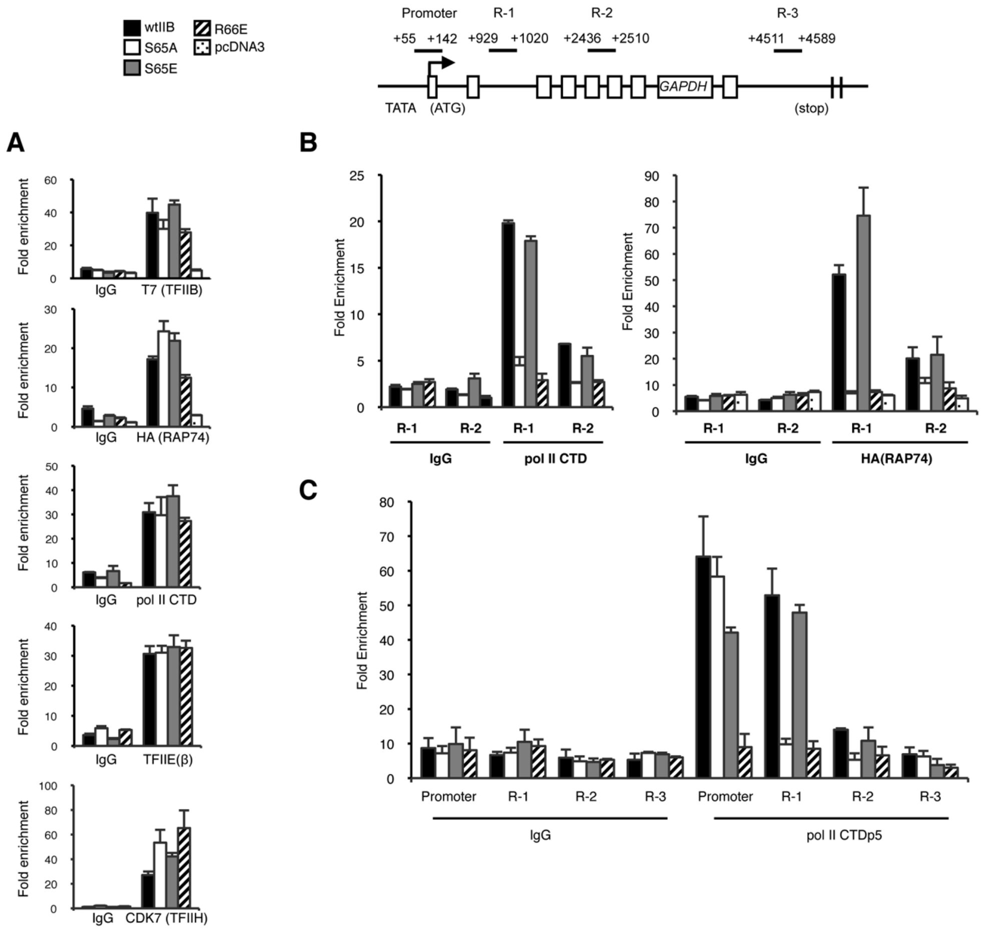

Figure S2. Analysis of PIC Assembly, Pol II Localization, and Pol II CTD Phosphorylation within the *GAPDH* Gene, Related to Figure 2

Data were generated and analyzed as in Figure 2, except that the subject was the *GAPDH* gene.

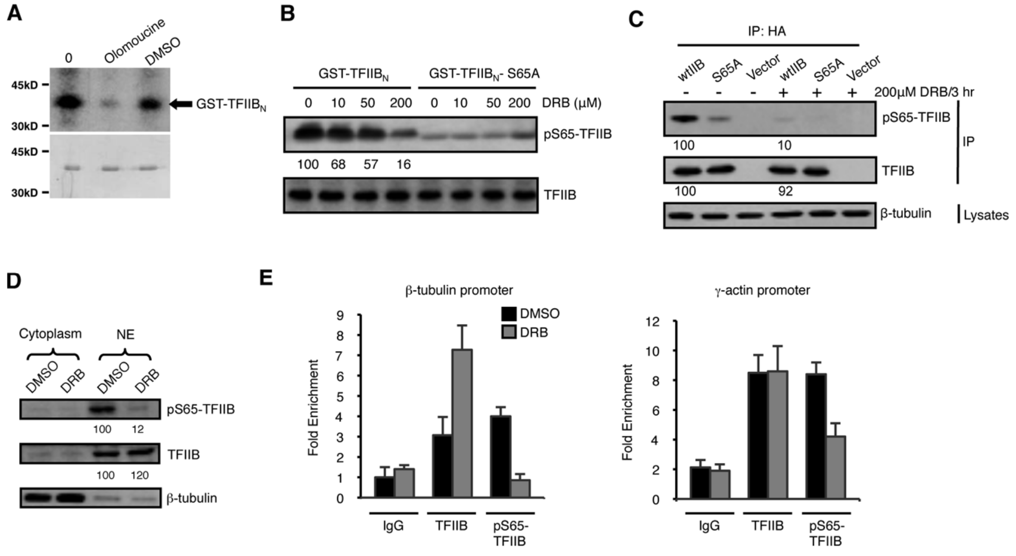

Figure S3. TFIIB Phosphorylation In Vitro and In Vivo Is Sensitive to CDK Inhibitors

(A) GST-TFIIB was incubated with crude HeLa cell nuclear extract in the presence of the CDK inhibitor olomoucine (100mM) where indicated. DMSO treatment was included as negative control. The kinase reaction was terminated and products analyzed as in Figure 1.

(B) GST-TFIIB$_N$ or GST-TFIIB$_N$ S65A was incubated with the 0.5M HeLa P11 fraction in the presence of DRB as indicated. Following the kinase reaction, the products were resolved by electrophoresis and then immunoblotted with anti-pS65-TFIIB antibodies (above) or anti-TFIIB antibodies (below).

(C) HEK293T cells were transfected with either pcDNA3 (Vector) or pcDNA3 driving expression of HA-tagged wild type TFIIB or TFIIB S65A. 48 hr after transfection, the cells were treated with 200μM of DRB where indicated for 3 hours at 37ºC. The ectopic TFIIB was immunoprecipitated from the cell lysates with anti-HA tag antibodies and the antibody-antigen complexes were resolved by SDS-PAGE. Western blot was performed with either anti-pS65-TFIIB antibody, anti-TFIIB antibody or anti-tubulin antibodies.

(D) HEK293T cells were treated with 200μM of DRB for 3 hours, and then separated into cytoplasmic and nuclear fractions. The samples were resolved by SDS-PAGE and western blots were performed with anti-TFIIB, anti-pS65-TFIIB, or anti-β-tubulin antibodies.

(E) HEK293T cells were treated with DRB or DMSO for 2 hours. ChIP was performed with anti-TFIIB antibodies, anti-pS65-TFIIB antibodies or control antibodies. qPCR was performed to analyze the β-*tubulin* and *γ-actin* promoters and are presented as the fold enrichment of the target promoter over the coding region of the same gene.

Supplemental Experimental Procedures

Plasmids and Proteins

G5AdML and pcDNA3 TFIIB have been described previously [12, 27]. The TFIIB derivatives S65A and S65E were produced by site-directed mutagenesis using the Stratagene QuikChange kit. GST-TFIIB and the derivatives GST-TFIIB (1-124), GST-TFIIB (124-316), and GST-TFIIB (1-124)-S65A were expressed and purified as previously described [26].

Cell Culture and FACS Sorting

Human embryonic kidney 293T (HEK 293T) cells and human osteosarcoma U2OS cells were grown in Dulbecco's modified Eagle's medium containing 10% fetal bovine serum at 37ºC. Transfection of HEK 293T cells was performed using calcium phosphate as described before [11]. Cells were harvested 48 hr after transfection, and the luciferase activity was measured with the Promega Luciferase kit and normalized to protein concentration based on Bradford protein assay. pSUPER driven expression of TFIIB shRNA was performed by the double transfection method described previously [12]. For FACS sorting, cells were trypsinized and collected by centrifugation at 1000 x g, the pellet was washed in cold PBS and resuspended in serum-free culture medium as a single cell suspension by filtering through a mesh filter (Filcon). The cells were analyzed and selected into GFP^+ populations in a fluorescence-activated cell sorter (BD Biosciences FACS Aria, FLS).

RNA Extraction and Quantitative RT-PCR Analysis

Total RNA was harvested using the Qiagen RNeasy Mini kit, and cDNA synthesis was performed with the Access reverse transcription kit (Promega). Samples were analyzed by quantitative real-time PCR using the $\Delta\Delta C_T$ analysis method. Quantitative PCR was performed in at least duplicate and from at least three independent experiments, using SYBR green Mastermix PCR reagent (Eurogentec) and a MJ research Chromo 4 machine (Bio-Rad). Results were analyzed with Opticon Monitor 3 software relative to input using the standard curve method. Values were normalized to 18S RNA level. Error bars show standard deviation of the mean.

Chromatin Immunoprecipitation Analysis

ChIP was performed with HEK293T cells grown in 100mm dishes, containing 1-1.5 x 10^7 cells. The cells were treated with 1% formaldehyde in phosphate-buffered saline for 10 minutes at room temperature before quenching with 0.125M glycine for 5 minutes. The cells were washed once with ice-cold phosphate-buffered saline, harvested, and then resuspended in SDS lysis buffer (50mM Tris pH 8.1, 10mM EDTA, 1% w/v SDS) containing protease inhibitors (1mM PMSF, Sigma complete protease inhibitor cocktail). Phosphatase inhibitors were included where indicated (1mM Sodium Vanadate, 30mM Sodium Fluoride and complete phosphatase inhibitor cocktail (Roche)). Lysates were briefly sonicated on ice for 10 seconds at medium output with a Misonix Sonicator XL-2000 first, and then samples up to 400µl were sonicated for 10 minutes at high power with a Diagenode sonicator to yield 200-600bp DNA fragments. The supernatant was diluted 1/10 in ChIP dilution buffer (16.7mM Tris pH 8.1, 167mM NaCl, 1.2mM EDTA, 1.1% Triton X-100, 0.01% SDS) and incubated overnight at 4ºC with either anti-T7 (Novagen), anti-HA (Santa Cruz), anti-CTD (Abcam), anti-CDK7 (Santa Cruz), anti-TFIIE (β; made by Scottish Diagnostics), anti-CTDp5 (Abcam), anti-CTDp2 (Abcam), anti-Ssu72 (Santa Cruz), anti-CstF64 (Santa Cruz), anti-phospho-S65-TFIIB (made by Eurogentec), anti-TFIIB (Santa Cruz), or non-

specific IgG antibodies (Upstate). 5 x 10^6 cells and 1μg of antibody were used per IP. Immune complexes were precipitated by incubation for 1 hour with protein-G-Sepharose beads that had been pre-blocked by incubation with 1mg/ml bovine serum albumin and 0.1mg/ml salmon sperm DNA. The chromatin was washed and decrosslinked as described previously [27]. DNA was cleaned using the Qiagen QiaQuick PCR cleanup kit. Quantitative PCR was performed as above using specific primers described in Supplementary information. Enrichment of target DNA (relative occupancy of $TFIIB_{T7}$, TFIIEβ, CDK7 (TFIIH), endogenous TFIIB, or phospho-TFIIB) is expressed as the ratio of the amount of promoter DNA bound over non promoter DNA. The enrichment of target DNA (relative association of CTD, CTDp5, CTDp2, $_{HA}$RAP-74, Ssu72, or CstF64) is expressed as the ratio of the amount of target DNA bound to the proteins of interest compared to nonspecific 18S DNA.

Immunoblot Analysis and Immunoprecipitation

Immunoblots were carried out with the primary antibodies directed against the following proteins: anti-TFIIB (sc-274, Santa Cruz), anti-HA (sc-805, Santa Cruz), anti-β-tubulin (sc-9104, Santa Cruz), anti-CstF64 (Santa Cruz), or anti-Ssu72 (Santa Cruz) and secondary antibodies were from Jackson ImmunoResearch. The pS65-TFIIB antibody was prepared by Eurogentec. The serum was first fractionated over a column containing the unphosphorylated peptide to remove general anti-TFIIB antibodies. The cleared serum was then fractionated over a column containing the phosphorylated TFIIB peptide. The column was washed extensively with Tris-buffered saline (0.1% v/v Tween) and the specific antibodies eluted with 50mM Glycine pH 2.5. The pS65-TFIIB antibody was used at a dilution of 1:250. Blots were visualized by enhanced chemiluminescence (GE Healthcare).

For immunoprecipitation, cells were lysed in the Triton lysis buffer (1% Triton X-100, 20mM Tris (pH 7.4), 137mM NaCl, 25mM sodium β-glycerophosphate, 2mM Sodium pyrophosphate, 2mM EDTA, 10% glycerol, 1mM phenylmethylsulfonyl fluoride, complete protease inhibitors (Sigma)). Where phosphatase inhibitors were included, this comprised of 1mM Sodium Vanadate, 30mM Sodium Fluoride and complete phosphatase inhibitor cocktail (Roche). The lysates were incubated with pre-bound antibody to protein G or A-Sepharose for 3 h at 4ºC. The beads were pelleted and washed three times in lysis buffer. Antibody-antigen complexes bound to the beads were eluted in SDS loading buffer, and analyzed by immunoblotting. Beads after the final wash were treated with lambda phosphatase (Cell Signaling) where required before immunoprecipitation according to the manufacturer's instructions.

Where indicated, cells were treated with 100nM calyculin-A for 30 minutes prior to lysis. Cells were lysed in Triton lysis buffer (0.5% Triton X-100) supplemented with protease- and phosphatase- inhibitors, 1 mg of cell extracts were precleared by adding 25µl Protein G Sepharose (Sigma) for 1 h at 4ºC. After centrifugation at 50 x g in a microfuge, the supernatant was incubated with 3-5µg of either anti-HA probe beads, protein G/A Sepharose-bound anti-TFIIB, anti-Ssu72, or anti-CstF64 for 3 h at 4ºC. The beads pellet was washed and resolved on SDS-PAGE and western blot analysis was performed with indicated antibodies.

P11 Fractionation of HeLa Cell Nuclear Extract and In Vitro Kinase Assay

A 5ml phosphocellulose P11 column (Whatman) equilibrated in 0.1M KCl-Buffer C (20mM Tris (pH 7.9), 10% glycerol, 1mM EDTA, 0.2mM phenylmethylsulfonyl fluoride, 20mM β-mercaptoethanol) was loaded with HeLa nuclear extract (10ml). Proteins were eluted with a

10ml step gradient from 0.1 to 1.0M KCl-Buffer C (at 0.1M, 0.3M, 0.5M, and 1.0M), each elution was dialyzed to 0.1M KCl-Buffer D (20mM HEPES (pH 8.0), 20% glycerol, 0.5mM EDTA, 0.2mM phenylmethylsulfonyl fluoride, 0.5mM dithiothreitol).

Recombinant GST-TFIIB (400ng) was added in a total volume of 200µl kinase reaction complex containing 1mM cold ATP, 10 μCi of γ-^{32}P-ATP, 100µl of P11 fraction, 25mM $MgCl_2$, 1mM Na_3VO_4, and 30mM NaF. Reactions were incubated at 30ºC for 30 minutes, the beads were then washed three times in 1M KCl-Buffer D and were boiled in SDS-loading buffer. Proteins were separated by 10% SDS-PAGE followed by Coomassie staining and phosphorylation was visualized by autoradiography.

Table S1. Primer Sequences

Gene		Primer sequences 5'-3' qRT-PCR
β-tubulin	Fwd:	TACCCTCCTCATCAGCAAGA
	Rev:	GGGTTCTGAAGCAAATGTCG
GAPDH	Fwd:	GGTCGTATTGGGCGCCTGGTCACC
	Rev:	CACACCCATGACGAACATGGGGGC
Amphiregulin	Fwd:	AGAGTTGAACAGGTAGTTAAGCCC
	Rev:	GTCGAAGTTTCTTTCGTTCCTCAG
γ-actin	Fwd:	GTTTGAGACCTTCAACACCC
	Rev:	CTTCATGAGGTAGTCGGTCAG
18S	Fwd:	GTAACCCGTTGAACCCCATT
	Rev:	CCATCCAATCGGTAGTAGCG
		**Primer sequences 5' – 3' ChIP**
β-tubulin	Promoter-Fwd:	TGCGGCTGACCAATAAAGAC
	Promoter-Rev:	TGCACGATTTCCCTCATGAT
	503 (Region 1)-Fwd:	TCAACGTGTACTACAATGAG
	503 (Region 1)-Rev:	CCAAAGCCGATTTAGTAGAG
	1372 (Region 2)-Fwd:	TACCCTCCTCATCAGCAAGA
	1372 (Region 2)-Rev:	GGGTTCTGAAGCAAATGTCG
	2154 (Region 3)-Fwd:	CCGAGAGCAACATGAATGAC
	2154 (Region 3)-Rev	GGCTGGGAGTGAATAAAGAG
		**Primer sequences 5' – 3' ChIP**
γ-actin	Promoter-Fwd:	GGAAAGATCGCCATATATGGAC
	Promoter-Rev:	TCACCGGCAGAGAAACGCGAC
	989 (Region 1)-Fwd:	GCTGTTCCAGGCTCTGTTCC
	989 (Region 1)-Rev:	ATGCTCACACGCCACAACATGC
	1651 (Region 2)-Fwd:	GTGACACAGCATCACTAAGG
	1651 (Region 2)-Rev:	ACAGCACCGTGTTGGCGT
	2549 (Region 3)-Fwd:	TCTGTCAGGGTTGGAAAGTC
	2549 (Region 3)-Rev:	AAATGCAAACCGCTTCCAAC
		**Primer sequence 5' – 3' ChIP**
GAPDH	Promoter-Fwd:	CTCCTGTTCGACAGTCAGC
	Promoter-Rev:	TTCAGGCCGTCCCTAGC
	929 (Region 1)-Fwd:	AAGTCAGGTGGAGCGAG
	929 (Region 1)-Rev:	TAAACCCACTTCTTTGATTTACCAGAG
	2436 (Region 2)-Fwd:	ATAGGCGAGATCCCTCCAA
	2436 (Region 2)-Rev:	TGAAGACGCCAGTGGAC
	4511 (Region 3)-Fwd:	AGATGTGTCAGGGTGACTTAT
	4511 (Region 3)-Rev:	TAGGTCCCAGCTACACGC
		**Primer sequences 5' – 3' ChIP**
18S gene	Fwd:	GTAACCCGTTGAACCCCATT
	Rev:	CCATCCAATCGGTAGTAGCG
